# Complete Genome Sequences of the Endophytic *Streptomyces* Strains EN16, EN23, and EN27, Isolated from Wheat Plants

**DOI:** 10.1128/genomeA.01342-16

**Published:** 2016-12-08

**Authors:** Christopher M. M. Franco, Ricardo Araujo, Eric Adetutu, Shanan S. Tobe, Sandeep Mallya, Bobby Paul, Kapaettu Satyamoorthy

**Affiliations:** a Medical Biotechnology, School of Medicine, Flinders University, Adelaide, Australia; b i3S, Instituto de Investigação e Inovação em Saúde and IPTIMUP, Institute Molecular Pathology and Immunology, University of Porto, Porto, Portugal; c School of Biological Sciences, Flinders University, Adelaide, Australia; d Department of Chemistry and Physics, Arcadia University, Glenside, Pennsylvania, USA; e School of Life Sciences, Manipal University, Manipal, India

## Abstract

The complete genome sequences of three endophytic *Streptomyces* species were compared. Strains EN16, EN23, and EN27 were isolated from surface-sterilized roots of wheat plants from South Australia. In field trials, these strains are effective in suppressing fungal root diseases of wheat when added as spore coatings to wheat seed.

## GENOME ANNOUNCEMENT

Actinobacteria are an important component of the plant microbiome, especially colonizing the roots (1, 2), with *Streptomyces* by far the most common endophytic actinobacterium (3). *Streptomyces* strains EN16, EN23, and EN27 were isolated as endophytes of wheat plants (4). Their spores when added as seed coatings colonized the wheat seedlings on germination (5) but did not significantly interfere with the indigenous endophytic populations within the wheat plants (6). They are capable of suppressing fungal pathogens, such as *Rhizoctonia solani, Pythium* spp., and *Gaeumannomyces graminis* var. *tritici*, and this treatment offers large advantages in controlling fungal root diseases in broadacre crops, resulting in higher grain yields in field trials (7, 8). Endophytic *Streptomyces* spp. act by promoting plant growth, facilitating nutrient acquisition, phytohormone production, antibiosis, and induction of systemic defense responses (7, 9). In the last instance, these species compete with plant pathogens and restrict pathogen colonization within the plant (10).

Here, we describe the complete genome sequences of *Streptomyces* sp. strains EN16, EN23, and EN27 obtained using the Illumina MiSeq sequencing platform (AGRF, Sydney, Australia). A total of 1.05 Gb of data were produced with next-generation sequencing technology for all three strains. The genome library contained different fragment length insertions (500 bp and 500 Mbp). The *de novo* assembly was performed using A5-miseq (11) and consisted of 236, 131, and 162 contigs for strains EN16, EN23 and EN27, respectively; the number of subsystems described was 443, 424, and 423 for the three strains, respectively, with a genome coverage of 69%. The Rapid Annotations using Subsystems Technology (RAST) server was used for gene prediction and annotation and the SEED viewer platform for the curation of genomic data (12).

The complete genomes of these three strains of *Streptomyces* sp. showed circular chromosomes. Strain EN16 presented 8,577,085 bp and a G+C content of 71.5%, the EN23 genome comprised 7,443,744 bp and a G+C content of 71.6%, and the genome of EN27 strain displayed 7,555,876 bp and a G+C content of 71.6%. The number of coding sequences in these strains ranged from 6,800 (for EN23) to 7,783 (for EN16); EN27 comprised 6,856 coding sequences, including sequences for the 13-kb plasmid described previously (13). The number of RNA genes reported was 85, 82, and 80 for EN16, EN23, and EN27, respectively.

A comparison of the *Streptomyces* genomes with the RAST database revealed the closest neighbors for EN16 and EN23 to be *Streptomyces griseus* subsp. *griseus* NBRC 13350 (genome ID455632.3); the EN27 genome was closer to *Streptomyces roseosporus* NRRL 11379 (genome ID457430.0). Further analysis of the 16S rRNA gene revealed the nearest-neighbor type strains for EN16 to be *Streptomyces fulvissimus* and *Streptomyces griseus*, and for EN23 and EN27, they are *Streptomyces badius* and *Streptomyces parvus*.

### Accession number(s).

These whole-genome shotgun projects for *Streptomyces* EN16, EN23, and EN27 have been deposited at DDBJ/ENA/GenBank under the accession numbers MJAF00000000, MJAI00000000, and MJAG00000000, respectively, and the corresponding versions described in this paper are MJAF01000000, MJAI01000000, and MJAG01000000.
